# Risk of GERD-Related Disorders in Obese Patients on PPI Therapy: a Population Analysis

**DOI:** 10.1007/s11695-018-3246-4

**Published:** 2018-05-01

**Authors:** Simon Erridge, Osama M. Moussa, Paul Ziprin, Ara Darzi, Sanjay Purkayastha

**Affiliations:** Department of Surgery and Cancer, Division of Surgery, Academic Surgical Unit, St. Mary’s Hospital, Imperial College London, 10th Floor QEQM, South Wharf Road, London, W2 1NY UK

**Keywords:** Obesity, GERD, Barrett’s esophagus, Esophageal carcinoma

## Abstract

**Background:**

Increasing prevalence of obesity has shown an associated increase in gastroesophageal reflux disease (GERD)-related diseases. Proton pump inhibitor (PPI) therapy has been demonstrated to reduce the incidence of such diseases. The study’s aim was to analyze the Clinical Practice Research Datalink (CPRD) to determine factors that increase the propensity of obese patients on PPIs to develop Barrett’s esophagus (BE) and esophageal carcinoma.

**Method:**

A case-control population study was carried out, including patients from the CPRD. Clinicopathological factors were extracted for each patient alongside clinical endpoints of GERD, BE, and esophageal carcinoma. Multivariate analysis was utilized to identify factors that increase the propensity to develop BE and esophageal carcinoma. Statistical significance was *p* < 0.050.

**Results:**

One hundred sixty five thousand nine hundred twenty nine obese patients on PPI treatment were identified up until July 2017. Median follow-up time was 119.0 months (range 11.3–1397.9 months). In patients with GERD, the following were associated with increased BE risk: age ≥ 60 years (OR = 1.197; *p* = 0.039), male (OR = 2.209; *p* < 0.001), H2 antagonists (OR = 1.377; *p* < 0.001), D2 antagonists (OR = 1.241; *p* = 0.008), and hiatus hernias (OR = 6.772; *p* = 0.017). The following were associated with increased risk of esophageal carcinoma: age (OR = 2.831; *p* = 0.031), male sex (OR = 3.954; *p* = 0.003), and hiatus hernias (OR = 12.170; *p* < 0.001). Only D2 antagonists (OR = 2.588; *p* = 0.002) were associated with increased risk of developing esophageal carcinoma in BE patients.

**Conclusions:**

In obese patients on PPI therapy for reflux, higher BMIs were not associated with increased risk of BE or esophageal carcinoma. Males, older patients, and those with hiatus hernias are at increased risk of developing BE and carcinoma. Failure of PPI monotherapy is predictive of future metaplasia and dysplasia.

**Electronic supplementary material:**

The online version of this article (10.1007/s11695-018-3246-4) contains supplementary material, which is available to authorized users.

## Introduction

Obesity is increasing in prevalence; global estimates believe that the number of individuals who are either overweight or obese is around 2.1 billion [1]. The metabolic complications of obesity (diabetes, hyperlipidemia, and hypertension) are well recognized [2–4]. Obesity is also shown, however, to correlate with an increase in gastroesophageal reflux disease (GERD)-related disorders, including Barrett’s esophagus (BE) and esophageal carcinoma [5]. The rising obesity epidemic is therefore presumed to contribute to the increased incidence in GERD-related disorders [5].

Abdominal obesity and body mass index (BMI) are proven risk factors for increasing esophageal reflux [5, 6]. Reflux of gastric contents and bile into the esophagus induces inflammation that can result in metaplasia and dysplasia of the esophageal epithelium [7]. Previous analysis of factors associated with GERD-related disorders additionally identifies genetic, demographic, behavioral, and co-morbid factors that increase the propensity of developing this spectrum of diseases [8, 9]. These however have not been studied within a uniquely obese population to elucidate which of these factors, if any, increase the progression of reflux-related disease and symptomology.

Proton pump inhibitors (PPIs) are an effective treatment for symptomatic relief from reflux esophagitis [10]. PPIs have also been demonstrated to effectively reduce the duration of esophageal acid exposure in obese patients, across all examined obesity classes [11]. This was confirmed clinically as PPIs negate any propensity to show progressive esophageal erosion or GERD. Despite this, it is still known that obese patients on PPI therapy still progress to develop GERD-related disorders [12].

The aim of this study was to perform a population analysis of obese patients on PPIs to elucidate any risk factors that increase the propensity to develop Barrett’s esophagus or esophageal carcinoma.

## Methods

### Study Design

A population-based case-control study of obese patients (BMI ≥ 30 kg/m^2^) who received PPI therapy was conducted utilizing the Clinical Practice Research Datalink (CPRD) in the UK. An analysis was conducted to identify potential factors that might influence the progression of this group of patients towards GERD, BE, or esophageal carcinoma.

The Clinical Practice Research Datalink is the largest clinical database in the UK, derived from a population of 8.5% of general practices. Campbell et al. have previously demonstrated the CPRD to be a representative sample of the national population [13]. The CPRD has also shown equivalent incident rates to other epidemiological analyses of the UK’s general population [14]. A median of 89% of reported cases within the CPRD are also confirmed on internal or external validation [14]. The data extraction collected patient visits between 1987 and 2017. Patients were followed up longitudinally until last appointment prior to data extraction, change of practice, or death.

### Patient and Data Selection

Patients were identified from the CPRD via clinical codes that were determined using a consensus approach. The initial step of this required the generation of definitions for the disease of interest. This was defined as patients with BMI ≥ 30 kg/m^2^ and had received PPI therapy by all authors. Subsequently, a list of clinical codes was generated between two authors (S.E. and O.M.). A comprehensive collection of synonyms was created for each variable. These were cross-matched against “medcodes” and descriptions provided by the CPRD for every disease, medication, and symptom using Stata (StataCorp 14, TX, USA). Data was extracted and cleaned in a systematic manner from the CPRD. Incidence of three clinical end points was isolated: GERD, BE, and esophageal carcinoma. For these, as well as BMI and PPI, dates of first and last patient encounter where these were present were also sought. Patients’ maximum-recorded BMI was used in assessing obesity status. These were categorized according to the World Health Organization classification: class I (30 kg/m^2^ ≤ BMI < 35 kg/m^2^), class II (35 kg/m^2^ ≤ BMI < 40 kg/m^2^), and class III (BMI ≥ 40 kg/m^2^) [15].

A number of demographic details were also obtained including gender, age, and marital status. The prevalence of previously identified modifiable risk factors was also extracted. This included smoking, use of non-steroidal anti-inflammatories (NSAIDS), aspirin, corticosteroids, bisphosphonates, dopamine-2 (D2) antagonists, hormone replacement therapy (HRT), or oral contraceptive pill (OCP). The presence of other associated diseases was also recorded, such as hiatus hernia, gastritis/peptic ulcer disease (PUD), diabetes mellitus, hypertension, and hyperlipidemia. Coding data was also analyzed to determine which patients also received a prescription for histamine-2 (H2) antagonists for concurrent control of acid symptoms, while already on PPI therapy—termed “dual therapy.”

Exclusion criteria included patients with a BMI ≥ 80 kg/m^2^ and/or aged ≥ 100 years old on first input into the CPRD to control for extreme variables that may account for incorrect coding.

### Statistical Analysis

Demographic variables were analyzed using descriptive analysis. Univariate analysis was utilized to determine prognostic factors for progression onto GERD-related disorders from initial PPI commencement date. A logistic regression model was used to calculate odds ratios (ORs), with their associated 95% confidence interval (95% CI). Multivariate analysis was conducted of factors where the ORs had a *p* value < 0.050 on univariate analysis. If one or fewer variables were found to have a *p* value < 0.050, multivariate analysis would be conducted with variables which had a *p* value < 0.100 to ensure that any potential prognostic factors were fully evaluated. Significance of multivariate analysis was determined using *p* value < 0.050. Missing values were excluded from analysis. Statistical analysis was conducted using Statistical Package for Social Sciences (SPSS) [IBM Statistics version 24 SPSS Inc., (Chicago, IL), USA].

## Results

One hundred sixty five thousand nine hundred twenty nine patients on the CPRD were identified with obesity on PPI treatment up until July 2017. The median follow-up time from date of starting PPI until database extraction or patient death was 119.0 months (range 11.3–1397.9 months). The median time between the first and last prescription for PPI therapy was 42.3 months (range 0.0–1397.2). Of this group, a number were diagnosed with GERD (*n* = 42,356), BE (*n* = 2119), and esophageal carcinoma (*n* = 60). Of these, some patients had GERD and BE (*n* = 934), GERD and esophageal carcinoma (*n* = 28), BE and esophageal carcinoma (*n* = 60), or all three diagnoses (*n* = 28). The demographic details of this cohort are detailed in full in Table 1.

**Table 1:** Demographic data

	PPI	GERD	BE	EC
Subjects (*n*)	165,929	42,356	2119	60
Age	53.2 ± 15.3	51.6 ± 14.3	58.7 ± 12.9	61.7 ± 12.2
Sex				
Male	57,775 (34.8%)	13,622 (32.2%)	1080 (51.0%)	44 (73.3%)
Female	108,147 (65.2%)	28,734 (67.8%)	1039 (49.0%)	16 (26.7%)
BMI at first recording	33.1 ± 6.2	32.6 ± 6.0	32.1 ± 5.0	32.4 ± 4.3
BMI at last recording	35.7 ± 6.5	35.6 ± 6.3	33.8 ± 5.7	30.5 ± 5.2
Max BMI	38.7 ± 6.6	38.5 ± 6.4	37.1 ± 5.6	36.9 ± 5.9
BMI category				
Class I	57,117 (34.4%)	14,410 (34.0%)	909 (42.9%)	29 (48.3%)
Class II	52,052 (31.4%)	13,727 (32.4%)	701 (33.1%)	21 (35.0%)
Class III	56,760 (34.2%)	14,219 (33.6%)	509 (24.0%)	10 (16.7%)
Hypertension	34,369 (20.7%)	9838 (23.2%)	525 (24.8%)	17 (28.3%)
Diabetes mellitus	47,082 (28.4%)	11,766 (27.8%)	648 (30.6%)	21 (35.0%)
Hyperlipidemia	14,318 (8.6%)	4327 (10.2%)	260 (12.3%)	7 (11.7%)
Tobacco use	42,433 (25.6%)	10,881 (25.7%)	422 (19.9%)	12 (20.0%)
Medication use				
Dual therapy	20,726 (12.5%)	8678 (20.5%)	493 (23.3%)	14 (23.3%)
NSAIDs	141,278 (85.1%)	36,429 (86.0%)	1685 (79.5%)	47 (78.3%)
Aspirin	57,703 (52.9%)	23,564 (55.6%)	1388 (65.5%)	45 (75.0%)
HRT	20,292 (12.2%)	7051 (16.6%)	219 (10.3%)	2 (3.3%)
OCP	7885 (4.8%)	2254 (5.3%)	137 (6.5%)	7 (11.7%)
Steroids	56,458 (34.0%)	16,143 (38.1%)	801 (37.8%)	28 (46.7%)
Bisphosphonates	11,507 (6.9%)	3211 (7.6%)	233 (11.0%)	9 (15.0%)
D2 antagonist	42,827 (25.8%)	15,520 (36.6%)	798 (37.7%)	37 (61.7%)
Gastritis/PUD	8356 (5.0%)	3247 (7.7%)	200 (9.4%)	3 (5.0%)
Hiatus hernias	18,931 (11.4%)	8900 (11.7%)	1146 (54.1%)	29 (48.3%)

*PPI* proton pump inhibitor, *GERD* gastroesophageal reflux disease, *BE* Barrett’s esophagus, *EC* esophageal carcinoma, *BMI* body mass index (kg/m^2^), *Dual therapy* histamine-2 receptor antagonist use during PPI therapy, *NSAIDs* non-steroidal anti-inflammatories, *HRT* hormone replacement therapy, *OCP* oral contraceptive pill, *D2 antagonist* dopamine-2 receptor antagonist, *PUD* peptic ulcer disease

### Prognostic Factors—GERD to BE

Table 2 outlines the variables that were evaluated for their effect on the likelihood of a patient with GERD developing BE in full. The following were all found to affect the development of BE: age ≥ 60 years old (OR = 1.425; *p* < 0.001), male sex (OR = 1.926; *p* < 0.001), class II obesity (OR = 0.47; *p* = 0.047), class III obesity (OR = 0.579; *p* < 0.001), hyperlipidemia (OR = 1.355; *p* = 0.003), tobacco use (OR = 0.763; *p* = 0.003), dual therapy (OR = 1.563; *p* < 0.001), NSAIDs (OR = 0.818; *p* = 0.043), aspirin (OR = 1.402; *p* < 0.001), HRT (OR = 0.776; *p* = 0.014), bisphosphonates (OR = 1.3000; *p* = 0.026), D2 antagonists (OR = 1.419; *p* < 0.001), gastritis/PUD (OR = 1.677; *p* < 0.001), and hiatus hernias (OR = 6.12; *p* < 0.001).

**Table 2:** Univariate analysis GERD to BE

Variable	Number	OR (95% CI)	*p* value
Age			
< 60 years old	29,562	1	
≥ 60 years old	12,599	1.425 (1.231–1.648)	< 0.001
Sex			
Female	28,630	1	
Male	13,531	1.926 (1.670–2.221)	< 0.001
BMI category			
Class I	14,331	1	
Class II	13,661	0.847 (0.719–0.998)	0.047
Class III	14,169	0.579 (0.483–0.695)	< 0.001
Hypertension			
Absent	32,369	1	
Present	9792	1.090 (0.926–1.282)	0.299
Diabetes mellitus			
Absent	30,450		
Present	11,711	0.977 (0.835–1.144)	0.774
Hyperlipidemia			
Absent	37,859	1	
Present	4302	1.355 (1.106–1.660)	0.003
Tobacco			
Non-smoker	31,323	1	
Smoker	10,838	0.763 (0.639–0.912)	0.003
Dual therapy			
Not prescribed	33,536	1	
Prescribed	8625	1.563 (1.337–1.827)	< 0.001
NSAIDs			
Not prescribed	5887	1	
Prescribed	36,274	0.818 (0.674–0.993)	0.043
Aspirin			
Not prescribed	18,726	1	
Prescribed	23,435	1.402 (1.206–1.631)	< 0.001
HRT			
Not prescribed	35,130	1	
Prescribed	7031	0.776 (0.634–0.949)	0.014
OCP			
Not prescribed	39,924	1	
Prescribed	2237	1.275 (0.966–1.681)	0.086
Steroids			
Not prescribed	26,100	1	
Prescribed	16,061	1.116 (0.966–1.290)	0.135
Bisphosphonates			
Not prescribed	38,969	1	
Prescribed	3192	1.300 (1.032–1.638)	0.026
D2 antagonists			
Not prescribed	26,745	1	
Prescribed	15,416	1.419 (1.230–1.637)	< 0.001
Gastritis/PUD			
No diagnosis	38,934	1	
Positive diagnosis	3227	1.677 (1.357–2.072)	< 0.001
Hiatus hernia			
No diagnosis	33,364	1	
Positive diagnosis	8797	6.107 (5.260–7.092)	< 0.001

Multivariate analysis confirmed that patients ≥ 60 years old (OR = 1.197; 95% CI 1.009–1.420; *p* = 0.039), male (OR = 2.209; 95% CI 1.846–2.644; *p* < 0.001), on dual therapy (OR = 1.377; 95% CI 1.160–1.635; *p* < 0.001), D2 antagonist use (OR = 1.241; 95% 1.057–1.458; *p* = 0.008), and having a hiatus hernia (OR = 6.772; 95% CI 1.410–32.529; *p* = 0.017) were at increased likelihood of developing BE.

### Prognostic Factors—GERD to Esophageal Cancer

Table 3 displays all factors analyzed to determine their relationship between developing esophageal cancer following GERD in full. The following were all found to have an effect: age ≥ 60 years old (OR = 4.284; *p* = 0.002), male sex (OR = 3.204; *p* = 0.010), class III obesity (OR = 0.166; *p* = 0.019), aspirin (OR = 3.770; *p* = 0.028), and hiatus hernias (OR = 12.928; *p* < 0.001).

**Table 3:** Univariate analysis GERD to esophageal carcinoma

Variable	Number	OR (95% CI)	*p* value
Age			
< 60 years old	29,656	1	
≥ 60 years old	12,674	4.284 (1.727–10.627)	0.002
Sex			
Female	28,721	1	
Male	13,609	3.204 (1.320–7.774)	0.01
BMI category			
Class I	14,402	1	
Class II	13,718	0.671 (0.262–1.720)	0.406
Class III	14,210	0.166 (0.037–0.744)	0.019
Hypertension			
Absent	32,500	1	
Present	9830	0.856 (0.307–2.391)	0.767
Diabetes mellitus			
Absent	30,569	1	
Present	11,761	1.285 (0.512–3.224)	0.593
Hyperlipidemia			
Absent	38,005	1	
Present	4325	0.879 (0.204–3.793)	0.863
Tobacco			
Non-smoker	31,458	1	
Smoker	10,872	1.413 (0.475–4.205)	0.534
Dual therapy			
Not prescribed	33,657	1	
Prescribed	8673	1.312 (0.497–3.462)	0.583
NSAIDs			
Not prescribed	5922	1	
Prescribed	36,408	0.464 (0.169–1.278)	0.138
Aspirin			
Not prescribed	18,779	1	
Prescribed	23,551	3.977 (1.164–13.592)	0.028
HRT			
Not prescribed	35,281	1	
Prescribed	7048	0.036 (0.000–4.754)	0.182
OCP			
Not prescribed	40,078	1	
Prescribed	2252	1.825 (0.423–7.870)	0.42
Steroids			
Not prescribed	26,197	1	
Prescribed	16,133	1.874 (0.776–4.525)	0.162
Bisphosphonates			
Not prescribed	39,119	1	
Prescribed	3211	1.884 (0.551–6.438)	0.312
D2 antagonists			
Not prescribed	26,824	1	
Prescribed	15,506	1.642 (0.693–3.890)	0.260
Gastritis/PUD			
No diagnosis	39,083	1	
Positive diagnosis	3247	0.575 (0.077–4.294)	0.589
Hiatus hernia			
No diagnosis	33,436	1	
Positive diagnosis	8894	12.928 (4.320–38.690)	< 0.001

Multivariate analysis found age (OR = 2.831; 95% CI 1.100–7.284; *p* = 0.031), male sex (OR = 3.954; 95% CI 1.603–9.755; *p* = 0.003), and hiatus hernias (OR = 12.170; 95% CI 4.044–36.627; *p* < 0.001) to all be associated with increased probability of developing esophageal cancer following GERD.

### Prognostic Factors—BE to Esophageal Cancer

Table 4 details all variables investigated that might influence rates of esophageal cancer in BE patients. Class III obesity reduced the probability of patients developing esophageal cancer compared to class I obesity (OR = 0.345; *p* = 0.019). D2 antagonist use was found to be positively associated with increased increase of esophageal carcinoma (OR = 2.927; *p* < 0.001).

**Table 4:** Univariate analysis BE to esophageal carcinoma

Variable	Number	OR (95% CI)	*p* value
Age			
< 60 years old	1111	1	
≥ 60 years old	1000	1.375 (0.776–2.439)	0.276
Sex			
Female	1034	1	
Male	1077	1.557 (0.744–3.261)	0.24
BMI category			
Class I	907	1	
Class II	698	1.727 (0.895–3.331)	0.103
Class III	506	0.345 (0.142–0.843)	0.019
Hypertension			
Absent	1589	1	
Present	522	1.677 (0.888–3.168)	0.111
Diabetes mellitus			
Absent	1468	1	
Present	643	0.586 (0.315–1.090)	0.091
Hyperlipidemia			
Absent	1851	1	
Present	260	0.848 (0.379–1.896)	0.688
Tobacco			
Non-smoker	1692	1	
Smoker	419	1.880 (0.861–4.102)	0.113
Dual therapy			
Not prescribed	1618	1	
Prescribed	493	1.059 (0.557–2.013)	0.861
NSAIDs			
Not prescribed	432	1	
Prescribed	1679	0.961 (0.477–1.935)	0.91
Aspirin			
Not prescribed	730	1	
Prescribed	1381	0.978 (0.517–1.849)	0.946
HRT			
Not prescribed	1892	1	
Prescribed	219	0.655 (0.156–2.749)	0.563
OCP			
Not prescribed	1976	1	
Prescribed	135	0.603 (0.212–1.718)	0.344
Steroids			
Not prescribed	1313	1	
Prescribed	798	0.743 (0.406–1.361)	0.336
Bisphosphanates			
Not prescribed	1878	1	
Prescribed	233	1.656 (0.796–3.446)	0.177
D2 antagonists			
Not prescribed	1316	1	
Prescribed	795	2.927 (1.631–5.254)	< 0.001
Gastritis/PUD			
No diagnosis	1911	1	
Positive diagnosis	200	0.509 (0.159–1.641)	0.258
Hiatus hernia			
No diagnosis	968	1	
Positive diagnosis	1143	0.602 (0.343–1.057)	0.077

Multivariate analysis found that D2 antagonist use was the only determinant factor that was associated with increased development from BE to esophageal carcinoma (OR = 2.588; 95% CI 1.426–4.695; *p* = 0.002).

## Discussion

This large-scale population analysis of obese patients on PPIs has elucidated some potential factors that may be associated with progression of GERD-related diseases. Despite PPI use, risk factors such as male sex, age, H2 antagonists, D2 antagonists, and hiatus hernias were additionally identified within this specific group of patients. Interestingly, no correlation was found between obesity beyond ≥ 35 kg/m^2^ and risk of progression to BE or esophageal carcinoma. Beyond this, the results of this study also indicate that the associated metabolic complications of obesity (diabetes, hypertension, and hyperlipidemia) are not associated with worsening reflux disease and that any effect of obesity is independent to this.

For patients with GERD, increased levels of obesity, notably class II and class II obesity compared to class I obesity, were not found to affect the rate of developing BE or esophageal carcinoma on multivariate analysis (*p* > 0.050). This is contradictory to a significant body of evidence that has linked obesity to increasing incidence of BE and esophageal carcinoma [16, 17]. However, the majority of previous evidence compares obese patients against normal weight controls, without subgroup analysis between obesity categories [6, 18]. Obesity is proposed to increase intra-abdominal pressure via abdominal obesity, resulting in increased reflux of gastric contents resulting in inflammation and potential progression to dysplasia and metaplasia [19]. Two mechanisms could be behind the lack of correlation seen. Firstly, increasing abdominal obesity, beyond a certain threshold, does not equate to increased gastric reflux. Secondly, PPI therapy may negate the negative effects of abdominal obesity; evaluation of PPI treatment by Sharma et al. found that reflux resolution is similar across BMI categories during PPI treatment [12]. A retrospective analysis additionally found that PPI therapy in obese patients is effective for resolution of gastric reflux [20]. This suggests that PPI therapy in this population may negate the potential impact of higher abdominal obesity in progression from GERD to BE or esophageal carcinoma. This highlights the importance of starting PPI treatment in patients with high BMI and reflux symptoms, or diagnosed with GERD-related disorders. These results may also be explained by a different hypothesis, that GERD is the intermediate step between obesity and the BE and esophageal carcinoma pathway. Subsequently, increasing obesity does not increase the risk of further progression but does increase the overall risk of patients with grade II/III obesity by increasing their initial risk of GERD. Therefore, PPI therapy will be important, in addition to seeking effective means to reduce weight.

An interesting factor highlighted in this analysis is that patients who required concurrent therapy with H2 antagonists were at increased risk from progression from GERD to BE (OR = 1.377). Dual therapy with both H2 antagonists and PPIs has been previously demonstrated to reduce acid reflux [21]. However, no studies have evaluated these patients over a prolonged follow-up period. These patients by definition have reflux disease that is refractory to PPI monotherapy. This suggests that the degree of reflux from which they suffer is worse either symptomatically, pathologically, or both. This long-term follow-up highlights that these patients are at increased risk of developing BE. This therefore indicates that while initiating dual therapy is beneficial for reducing esophageal acidity, these patients need closer follow-up to ensure compliance with medication and resolution of reflux. This is further highlighted by the fact that patients who were started on D2 antagonists, such as metoclopramide or domperidone, were also at increased risk of developing BE (OR = 1.241) and then subsequently esophageal carcinoma (OR = 2.588). D2 antagonists can be used to increase gastric motility to help reduce esophageal acidity and again may indicate more severe reflux therefore increasing risk of serious GERD-related conditions. This data, however, is confounded by its more common use as an anti-emetic, particularly in cancer patients. All together, these pieces of evidence do suggest that increased follow-up of patients with PPI-refractory reflux is important to reduce progression to metaplasia and subsequently dysplasia.

Hiatus hernia was shown to be associated with the greatest risk of BE (OR = 6.772) and esophageal carcinoma (OR = 12.170). Hiatus hernias have previously been shown to increase the risk of these conditions via increasing reflux of gastric contents into the esophagus [22]. Gastric fundoplication is effective in significantly reducing acid reflux in an obese cohort [23]. It provides improved resolution of reflux compared to PPI therapy for GERD with or without an associated hiatus hernia [24, 25]. Gastric bypass operations for morbid obesity have also been shown to reduce acid reflux, in addition to reducing BMI and metabolic conditions [26]. Future studies are required to evaluate whether a hybrid hiatus hernia repair and gastric bypass would also be a suitable therapeutic modality. In contrast to gastric bypasses, sleeve gastrectomy can aggravate GERD postoperatively [27]. Surgical therapy has been shown to improve patient quality of life, induce regression of metaplasia, and reduce progression to esophageal carcinoma over long-term evaluation [28]. It is therefore important that applicable patients are able to access appropriate surgical therapy to alleviate the consequences of raised gastric reflux. This includes patients with a hiatal hernia and those who fail PPI therapy. It is also important that candidates for bariatric surgery with GERD are advised to avoid sleeve gastrectomy as this may worsen their symptoms.

This study, despite benefiting from the analysis of a large population, is still subject to limitations. Any analysis from a database is reliant upon correct and timely coding of all clinical parameters. This is unable to be confirmed on an individual patient basis due to the inability to access original patient files. Subsequently, this may result in a discrepancy between clinical codes and actual patient disease or medication. Moreover, the length of follow-up within this study, despite a median of 119.0 months, may not be sufficient to capture all cases of BE or esophageal carcinoma within this cohort as the progression of both these diseases from GERD is typically over the course of many years. However, any misclassification or lack of capture would bias the results towards a non-significant difference. Additionally, selection bias is minimized as all included patients are derived from a database that has been previously validated as representative of the national population [13]. While the CPRD gives a reliable estimate of patient BMI across many years, factors such as waist circumference, which are infrequently measured, are difficult to include in analyses. Additionally, compliance with medication cannot be assessed reliably. Finally, due to clinical coding, it is not possible to evaluate all possible risk factors that may play a role in the pathogenesis of GERD-related conditions. The two most notable of these are ABO blood group and alcohol consumption, which have both been shown to be associated with said conditions [29, 30]. These are 2 variables that are not reliably assessed frequently enough in general practice clinics to be used with any certainty in this analysis. Despite this, analysis of the CPRD is still the most reliable way of conducting a retrospective analysis of a population size of this magnitude in the UK.

### Conclusions

This large population analysis of obese patients on PPI therapy has demonstrated that increasing BMI beyond an obese baseline does not significantly contribute to the development of BE and or esophageal carcinoma. Males, older patients, persons who fail PPI monotherapy, and those with hiatus hernias are at most increased risk of developing BE and carcinoma. These are important factors to consider when deciding which patients require most intensive follow-up or require appropriate interventions such as anti-reflux surgery or starting dual pharmacological therapy.

## Electronic Supplementary Material

ESM 1 (PDF 514 kb)
